# Anthocyanin Composition and pH Correlate with Berry Skin Color Across Diverse Grape Germplasm

**DOI:** 10.3390/foods15122242

**Published:** 2026-06-22

**Authors:** Fei Peng, Weichu Ouyang, Wenting Chen, Feixiong Luo, Yanshuai Xu, Guoshun Yang, Jun Tan

**Affiliations:** College of Horticulture, Hunan Agricultural University, Changsha 410128, China; pengf2001@126.com (F.P.); m15084992216@163.com (W.O.); slaizb123456@163.com (W.C.); luofx1988@hunau.edu.cn (F.L.); yx56@hunau.edu.cn (Y.X.); guoshunyang@aliyun.com (G.Y.)

**Keywords:** berry skin color, anthocyanidin stability, pH, CIE Lab color parameters, multivariate analysis, *Vitis davidii*

## Abstract

Berry skin color CIE parameters, pH and anthocyanidin profiling of 46 grape accessions were investigated using CIE Lab system, pH measurement and anthocyanidin profiling. CIE parameters separated the samples into three groups: yellowish-green, pinkish-red, and purplish-black, and principal component analysis confirmed clear clustering, with the first two components explaining 99.1% of the variance. After anthocyanidin analysis, cyanidin was detected in all samples, whereas trace-level pelargonidin derivatives were identified by UPLC-MS/MS. Total anthocyanidin content was insufficient to evaluate the quality of berry color, but anthocyanidin composition and relative proportions showed a stronger association with color classification. Yellowish-green berries were enriched in cyanidin, while purplish berries contained more malvidin- and cyanidin. Multivariate analysis identified cyanidin, malvidin, and peonidin derivatives as the main drivers of berry skin color variations. Skin homogenate pH ranged from 3.36 to 4.63 and it was lower in wild grape relatives. Correlation analysis indicated that pH was associated with color parameters. Species-related differences in anthocyanidin glycosylation and acylation were evident, and mono/diglucosides may have potential effects on skin color. Overall, skin color appears to depend on anthocyanidin composition, relative proportions, and pH, offering a chemical basis for grape breeding and fruit quality evaluation.

## 1. Introduction

Grape (*Vitis vinifera*) is one of the most economically important fruit crops cultivated worldwide, and berry skin color is a key trait determining both market value and consumer acceptance. For fresh consumption as well as for processing, color attributes such as lightness, hue, and saturation are widely used as critical indices for quality evaluation, as they directly influence visual appeal and product grading [1,2,3]. The formation of berry skin color is a complex process regulated by multiple factors, among which anthocyanin biosynthesis and physicochemical conditions play central roles [4,5]. A clearer understanding of this regulatory framework is therefore essential for improving grape quality and guiding its genetic improvement strategies.

Anthocyanidins, the aglycone form of anthocyanins, are water-soluble flavonoid pigments responsible for red, purple, and blue coloration in plant tissues. Six major anthocyanidin monomers—delphinidin, cyanidin, petunidin, pelargonidin, peonidin, and malvidin—are commonly found in plants, predominantly in glycosylated forms [6,7]. As the biochemical foundation of fruit coloration, the relative composition and total abundance of anthocyanidins, along with structural modifications including methylation and acylation, strongly influence color hue, resulting in phenotypic variation from bright red to deep purple or blue-black [8,9].

While anthocyanidin composition forms the primary biochemical basis for berry coloration, the final visual expression of color is also tightly regulated by the physicochemical properties of the vacuolar environment, most notably pH. pH is a critical physicochemical factor affecting color expression in grape berry skins. Changes in pH value alter the protonation state and molecular conformation of anthocyanidins, thereby modifying their visible color. Under acidic conditions, anthocyanidins primarily exist as stable flavylium cations, producing red coloration. Although the pH range in grape berry skins is typically acidic, it is known from in vitro studies [10,11,12] that as pH increases, anthocyanidin structure transforms toward quinoidal base and its chalcone forms occur, leading to a shift toward bluish hues. Together, anthocyanidin composition and pH define a fundamental regulatory axis governing berry skin color.

Although the individual roles of anthocyanidins and pH in grape berry skin coloration have been investigated, previous studies have notable limitations. Some have focused on temporal changes in anthocyanidin accumulation and color development within single cultivars [13], while others have compared anthocyanidin profiles across multiple cultivars, with some studies involving 15 or more cultivars [14,15]. These studies have underscored the importance of genetic background in berry skin color differentiation. However, research on pigment identification in lesser-known grape species or interspecific hybrids remains scare, and no study has systematically integrated anthocyanidin composition, pH and skin color across a broad germplasm panel including wild grape relatives.

Moreover, while anthocyanin profiles or pH have often been studied in relation to color independently, their combined analysis within a single, diverse germplasm set is rare. To fill these research gaps, this study innovatively: (1) analyzing 46 accessions covering *V. vinifera* cultivars, interspecific hybrids, and wild grape relatives; (2) employs complementary analytical techniques (HPLC for anthocyanidin aglycones and UPLC-MS/MS for intact anthocyanins); and (3) systematically correlated chemical profiles with instrumental skin color parameters and skin pH measurements. This comprehensive approach aims to provide a holistic understanding of the chemical determinants of berry skin color.

Therefore, the aims of this study were: (i) to quantitatively classify berry skin color across the diverse germplasm using CIE Lab color parameters; (ii) to investigate the associations between anthocyanidin composition, pH, and berry skin color at a multi-genetic background scale by integrating instrumental color measurements, pH determination, and anthocyanidin profiling. Identify the anthocyanidin types most strongly correlated with specific color axes (e.g., a*, b*) through correlation analysis and OPLS-DA; (iii) to characterize the chemical compounds responsible for grape skin color diversity, with a focus on key anthocyanins in wild grape relatives, including some rarely reported anthocyanins.

## 2. Materials and Methods

### 2.1. Chemicals and Materials

Delphinidin chloride (≥98%, 5 mg), petunidin chloride (≥98%, 5 mg), pelargonidin chloride (≥98%, 5 mg) and peonidin chloride (≥98%, 5 mg) were obtained from Jingcui Tiancheng Pharmaceutical Technology Co., Ltd. (Chengdu, China). Cyanidin chloride (≥98%, 20 mg) and malvidin chloride (≥97%, 5 mg) were purchased from Yuanye Bio-Technology Co., Ltd. (Shanghai, China). Ethanol (HPLC) was supplied by Tedia High Purity Solvents Co., Ltd. (Anqing, China). Methanol and acetonitrile were HPLC grade and purchased from Fudun Science and Technology Co., Ltd. (Wuhan, China). Phosphoric acid and formic acid were obtained from Sigma-Aldrich (St. Louis, MO, USA). Hydrochloric acid (guaranteed reagent grade) was supplied by Xinyang Chemical Reagent Factory (Xinyang, China). Purified bottled water was obtained from Zhongwo Water Environmental Protection Technology Co., Ltd. (Changsha, China).

### 2.2. Samples

A total of 46 accessions were selected to represent diverse genetic backgrounds, a wide color ranges from yellowish-green to purplish-black, and locally distinctive previously overlooked accessions, based on the availability of the research group’s germplasm resources and the sampling period. They comprised 22 *V. vinifera* cultivars, 16 interspecific hybrids and 8 wild grape relatives, collected from different regions of China. Detailed information for each accession is provided in Table S1. To minimize variation in maturity among accessions harvested from diverse locations, all berries were collected at commercial maturity as defined by soluble solids content.

Immediately after harvest, the fresh berries were subjected to colorimetric measurement. Subsequently, the skin tissues were excised and pooled to form a single composite sample per accession, then frozen in liquid nitrogen, ground into a fine powder, sealed in centrifuge tubes, and stored at −80 °C until further HPLC and UPLC-MS/MS analysis.

### 2.3. Color and pH Measurement

Chromaticity measurements were conducted on freshly harvested grape skins with uniform coloration and consistent fruit hue according to previously reported methods [16]. Grape skin color parameters (L*, a*, and b*) were measured using a 3nh NR110 colorimeter (Sanenshi Technology Co., Ltd., Shenzhen, China). The instrument employs an 8°/d geometry (8° illumination with diffuse reflectance reception) and features a Φ4 mm measuring aperture. It is fitted with a conical measuring tip to accommodate curved samples. The illuminant used is D65 (standard daylight), and a calibration against a standard white plate is conducted prior to each measurement. This colorimeter model is broadly applicable for color measurements on irregular surfaces, such as fruits and food products. For each of the 46 accessions, ten berries with uniform coloration were selected from the top, middle, and bottom sections of different clusters, and each berry was measured at three different positions (top, middle, and bottom).

For the determination of skin homogenate pH, berry samples were systematically collected from the top, middle, and bottom sections of multiple clusters (*n* ≥ 3 clusters per accession). Skin tissues from these berries were pooled to form a composite sample for each accession. Following the method described by Saarniit et al. [17], the pooled skin tissues were homogenized with distilled water at a precise 1:1 (*w*/*v*) dilution ratio prior to pH measurement. Then, a PHS-3C pH meter (Leici Instrument Co., Ltd., Shanghai, China) was used for determination. Measurements were performed in triplicate for each accession.

### 2.4. Anthocyanidin Analysis by HPLC

#### 2.4.1. Sample Preparation

Sample preparation was adapted from the method described by Liang et al. [18], with minor modifications. Briefly, 0.5 g of sample powder, prepared as described in Section 2.2, was extracted with 10 mL of an acidified ethanol-water solution (ethanol:hydrochloric acid:water = 2:1:1, *v*/*v*/*v*). The mixture was subjected to ultrasonic extraction for 30 min, followed by incubation in a boiling water bath for 1 h. After rapid cooling to room temperature, the extract was centrifuged, and the supernatant was collected and filtered through a 0.22 µm PTFE syringe filter and transferred to amber glass vials for subsequent HPLC analysis. All extractions and measurements were performed in triplicate.

#### 2.4.2. HPLC Analytical Conditions

A Waters Alliance high-performance liquid chromatography (HPLC) system equipped with an autosampler and a UV-visible detector (Waters Corporation, Milford, MA, USA), was applied for quantity analysis. Separation was performed on a WondaSil-C18 column (250 mm × 4.6 mm, 5 μm; Shimadzu Corporation, Kyoto, Japan). The mobile phase consisted of solvent A (0.2% phosphoric acid in water) and solvent B (acetonitrile), delivered at a constant flow rate of 1.2 mL∙min^−1^. The gradient program was as follows: 0–2 min, solvent A decreased from 90% to 81%; 2–11 min, solvent A decreased from 81% to 78%; 11–12 min, solvent A increased from 78% to 90%; and 12–20 min, solvent A maintained at 90%. The column temperature was set at 40 °C, the injection volume was 10 μL, and anthocyanidin detection was conducted at 530 nm.

Anthocyanidins were identified by comparing retention times with those of authentic standards. Quantification was carried out using external standard calibration curves prepared from mixed standards at five concentration levels in 10% hydrochloric acid-methanol. Sample extracts were analyzed under the same chromatographic conditions.

### 2.5. Anthocyanin Analysis by UPLC-MS/MS

#### 2.5.1. Sample Preparation

For the UPLC-MS/MS analysis, seven purple-black grape accessions with high anthocyanin content were selected for analysis including *Vitis vinifera* (‘Cabernet Sauvignon’), Euro-American hybrids (‘Summer Black’ and ‘Lanbaoshi’), and wild grape relatives (‘Xiangci No. 1’, ‘Xiangci No. 3’, ‘Xiangci No. 4’ and Adenostoma grape).

50 mg of berry skin powder, which was prepared as described in Section 2.2, was extracted with 500 µL of 50% (*v*/*v*) aqueous methanol containincg 0.1% hydrochloric acid. The mixture was vortexed for 5 min and sonicated for 5 min at ambient temperature. After sonication, the mixture was centrifuged at 12,000 rpm for 3 min at 4 °C. The supernatant was collected, and the extraction was repeated once under the same conditions. The combined supernatants were filtered through a 0.22 µm PTFE syringe filter and transferred to amber glass vials for subsequent UPLC-MS/MS analysis.

#### 2.5.2. UPLC-MS/MS Analytical Conditions

An ultra-performance liquid chromatography (UPLC) system (Sciex, Framingham, MA, USA) coupled with a QTRAP 6500+ triple quadrupole mass spectrometer (Sciex, Framingham, MA, USA) was used for analysis. Chromatographic separation was achieved on an ACQUITY BEH C18 column (2.1 mm × 100 mm, 1.7 μm; Waters Corp., Milford, MA, USA) at a flow rate of 0.35 mL min^−1^. The mobile phase consisted of solvent A (0.1% formic acid in water) and solvent B (0.1% formic acid in methanol). The gradient program was: 0.0 min, 5% solvent B; 6.0 min, 50% solvent B; 12.0 min, 95% solvent B; 12.0–14.0 min, held at 95% solvent B; 14.0 min, returned to 5% solvent B; and 14.0–16.0 min, re-equilibrated at 5% solvent B. The column temperature was maintained at 40 °C, and the injection volume was 2 μL.

Mass spectrometric analysis was carried out using an electrospray ionization (ESI) source operating in positive ion mode, with a source temperature of 550 °C and an ion spray voltage of 5500 V. The curtain gas (CUR) pressure was set to 35 psi. For the QTRAP 6500+ system, ion transitions were optimized by adjusting declustering potential (DP) and collision energy (CE).

Qualitative identification of anthocyanins was achieved by matching MS/MS spectra with those in the Metware Database (MWDB) constructed from authentic standards. Absolute quantitative analysis was conducted in multiple reaction monitoring (MRM) mode. In MRM, precursor ions were selectively filtered by the first quadrupole, fragmented in the collision cell, and specific product ions were monitored by the third quadrupole, thereby minimizing background interference and ensuring high quantification accuracy and reproducibility. Chromatographic peak integration and quantification were performed using external standard calibration curves.

**Figure 1:**
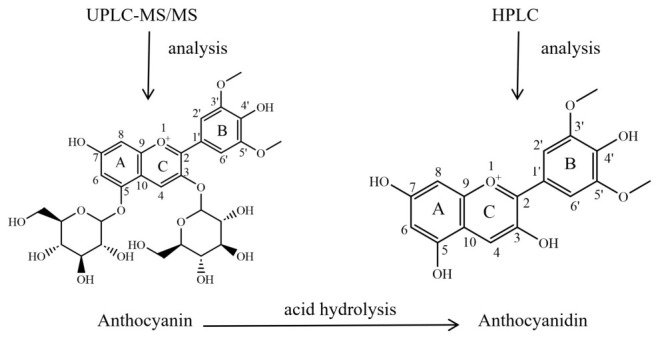
Schematic illustration of the structural relationship between anthocyanins (intact glycosides, detected by UPLC-MS/MS) and anthocyanidins (aglycones, detected by HPLC after acid hydrolysis). Malvidin-3,5-O-diglucoside and malvidin are used as representative examples.

Based on the methods above, anthocyanidins (aglycones) were quantified by HPLC after acid hydrolysis, which reflect the core structures of anthocyanins; while intact anthocyanins (glycosides) were analyzed by UPLC-MS/MS without acid hydrolysis, which represent the native compound structure in grape skins (Figure 1).

### 2.6. Data Processing and Multivariate Statistical Analysis

Data processing and bar chart generation were performed using Origin 2021 (OriginLab Corp., Northampton, MA, USA). Statistical analyses were conducted using SPSS Statistics 26 (IBM Corp., Armonk, NY, USA). Heat map visualization was carried out using TBtools II software (https://github.com/CJ-Chen/TBtools-II, accessed on 24 May 2026). Principal component analysis (PCA) and orthogonal partial least-squares discriminant analysis (OPLS-DA) were carried out using SIMCA 14.1 (Sartorius AG, Göttingen, Germany). All measurements are expressed as mean ± standard deviation (SD).

## 3. Results

### 3.1. Cultivar-Dependent Variation in Grape Berry Skin Color

**Figure 2:**
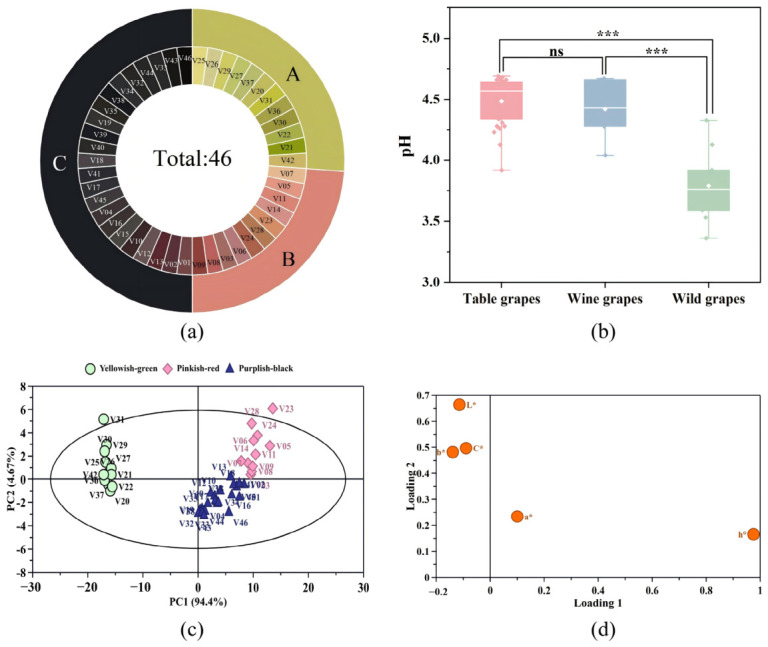
Skin color classification and skin homogenate pH characteristics of 46 grape accessions. (**a**) Nested circular plot illustrating the skin color distribution of 46 grape accessions, classified into yellowish-green (A), pinkish-red (B), and purplish-black (C) groups. (**b**) Box plots showing pH values of grape skin homogenate from grape cultivars (ns, not significant; *** *p* < 0.001). (**c**) PCA score plot based on CIE Lab color parameters of grape skins. (**d**) PCA loading plot indicating the interaction of individual color parameters.

Based on visual assessment and CIE lab colorimetric measurements (Figure 2a) combined with visual assessment, the 46 grape germplasm resources were classified into three color groups: yellowish green (Group A, *n* = 12, L* > 30, a* < 0.5, b* > 4), pinkish red (Group B, *n* = 11, L* > 25, a* > 1, b* ≈ 0), and purplish black (Group C, *n* = 23, L* < 27, a* ≈ 0 or >1, b* < 0).

Principal component analysis (PCA) of CIE Lab color parameters (L*, a*, b*, C*, and h°) showed the first two principal components accounted for 99.1% of the total variance (Figure 2c), indicating these parameters captured most of color variation among samples. Grape accessions clustered clearly according to color group in the score plot. The PCA loading plot (Figure 2d) revealed that L* (lightness), b* (yellowness), and C* (chroma) contributed strongly and positively to the separation of yellowish-green grapes, consistent with their brighter, more yellowish appearance. In contrast, a* (red-green coordinate) was positively associated with the pinkish-red and purplish-black groups, reflecting their red-purple coloration. These loading patterns align well with the observed visual color differences among the three groups.

Data were initially examined for normality (Shapiro–Wilk test) and homogeneity of variances (Levene’s test). For a*, which met both assumptions (*p* > 0.05), one-way ANOVA with LSD post hoc analysis was applied. For L*, b*, C*, and h°, which satisfied normality but showed unequal variances (*p* < 0.05), Welch’s ANOVA followed by Games–Howell post hoc tests were used. A *p*-value < 0.05 was considered statistically significant. Significant differences (*p* < 0.05) were detected for all color parameters among the three groups (Table 1). Yellowish-green grapes exhibited the highest L*, b*, and C* values, reflecting higher brightness and translucency, whereas purplish-black grapes showed the lowest L*, b*, and C*, consistent with darker skin coloration. These results confirmed that the diversity of berry skin colors can be quantitatively distinguished using CIE Lab parameters. However, the pattern identified in this study significantly differs from those reported by Campbell et al. [19], who found that only L* and h° traits could distinguish between colored and non-colored berries. Given that there were 46 accessions in our study, including table grapes, wine grapes and wild grape relatives, this finding provides a broader and more comprehensive basis than those reported exclusively in muscadine berry populations. Notably, the h° values of yellowish-green cultivars were significantly lower than those of other color groups. However, due to differences in color measurement instruments, absolute values should not be directly compared numerically. The discrepancies between our results and those of Liang et al. primarily reflect variations in overall color trends rather than direct numerical contradictions.

### 3.2. Analytical Method Validation for Anthocyanidin Determination by HPLC

The anthocyanidin content in grape skin samples was determined using a validated HPLC method following the procedure described in Section 2.4. The chromatographic conditions and comprehensive method validation results are presented below.

#### 3.2.1. Chromatographic Conditions and System Suitability

**Figure 3:**
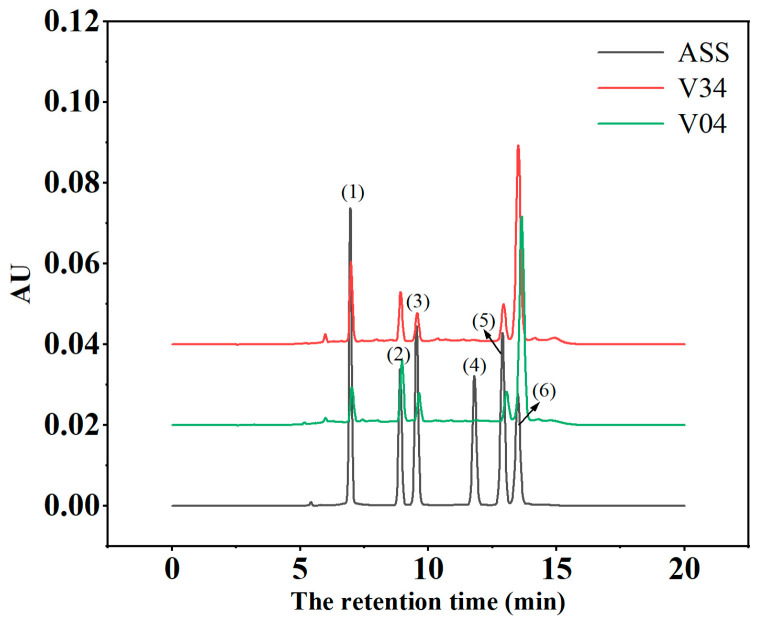
HPLC chromatogram of a mixed anthocyanidin standard solution (10 mg∙L^−1^) and two grape skin samples. The peaks correspond to delphinidin (1), cyanidin (2), petunidin (3), pelargonidin (4), peonidin (5), and malvidin (6). ASS refers to anthocyanidin standard solution; V34 refers to the skin sample of ‘Cabernet Sauvignon’; V04 refers to the skin sample of ‘Heimeiren’.

A representative HPLC chromatogram of a mixed standard solution containing six anthocyanidin aglycone (each at 10.0 mg∙L^−1^), and two grape skin samples was shown in Figure 3. The six target compounds were well-separated under the established gradient conditions. No interfering peaks were observed in the blank sample injections, confirming the specificity of the method.

#### 3.2.2. Linear Calibration and Sensitivity

Linear calibration curves were established for each anthocyanidin over the concentration range of 0.1 to 50 mg∙L^−1^. The calibration data are summarized in Table 2. All six compounds exhibited excellent linearity, with correlation coefficients (R^2^) exceeding 0.9999. The high R^2^ values and consistent response across the wide concentration range confirm the method’s suitability for quantitative analysis.

The sensitivity of the method was evaluated by determining the limit of detection (LOD) and limit of quantification (LOQ) for each anthocyanidin, as detailed in Table 3. LOD values ranged from 0.0025 mg∙L^−1^ for delphinidin to 0.0050 mg∙L^−1^ for the remaining anthocyanidins. The corresponding LOQs, based on a signal-to-noise ratio of 10, ranging from 0.1660 to 0.3340 mg∙kg^−1^ in the sample matrix. These results demonstrate the high sensitivity of the developed HPLC method.

#### 3.2.3. Instrument Precision and Method Stability

The instrumental precision was evaluated by six consecutive injections of a 1.0 mg∙L^−1^ anthocyanidin mixed standard solution. As detailed in Table 3, the relative standard deviation (RSD) of peak areas for all compounds was below 1.2%, with most below 0.5%, indicating excellent repeatability of the HPLC system. The stability of the sample solution was tested by repeatedly injecting a ‘Yeniang No. 2’ sample extract at 2 h intervals over a 24 h period. All RSDs of quantifiable anthocyanidins were below 1.0%, confirming that the analytes were stable in the prepared sample solution under the analytical conditions for at least 24 h.

#### 3.2.4. Method Accuracy

The accuracy of the method was evaluated through recovery experiments by spiking known amounts of anthocyanidin standards into pre-analyzed grape skin samples at three concentration levels: low (0.5 mg∙L^−1^), medium (1.0 mg∙L^−1^), and high (10 mg∙L^−1^). As presented in Table 3, the recoveries ranged from 81.85% to 119.1% across all spiking levels. Most recoveries fell within the acceptable range of 80–120%, supporting the acceptable accuracy of the method for quantifying anthocyanidins in grape skin matrices.

### 3.3. Correlations Between Berry pH and Anthocyanidin Composition Profiles

Grape berry skin homogenate pH values ranged from 3.36 to 4.63 (Figure 2b), in agreement with previously reported values for grape tissues [20,21]. For pH, data met the assumptions of normality and homogeneity of variances (*p* > 0.05); thus, one-way ANOVA with LSD post hoc analysis was applied. No significant difference was observed between *V. vinifera* cultivars and interspecific hybrids, whereas wild grape relatives exhibited significantly lower pH values, likely reflecting the absence of artificial selection for reduced acidity.

**Figure 4:**
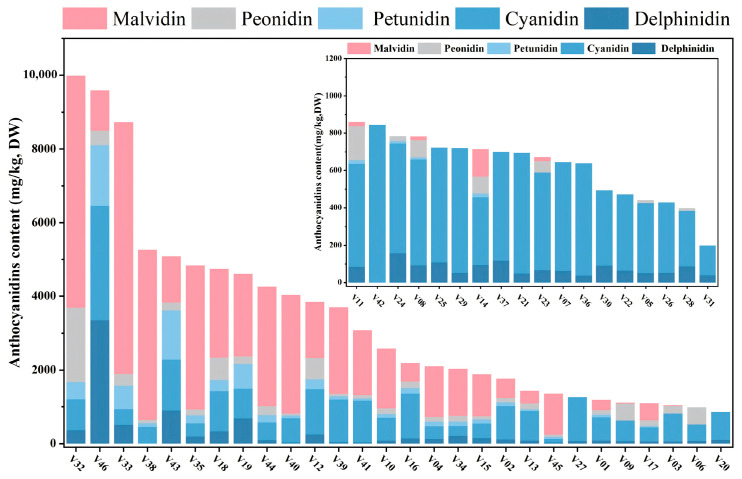
Anthocyanidin composition and contents in berry skins of 46 grape accessions. Stacked bar chart showing anthocyanidin contents in grape skins on a dried weight basis. To improve clarity and avoid overcrowding the main panel, accessions with low anthocyanidin levels are presented solely in the inset.

Anthocyanidin composition and content quantified by HPLC, varied substantially among grape accessions (Figure 4). Cyanidin was detected in all samples, while trace-level pelargonidin derivatives were identified by UPLC-MS/MS. Although total anthocyanidin content generally increased as skin color deepened, several yellowish-green cultivars exhibited higher total anthocyanidin content than some pinkish cultivars, with over 80% of the anthocyanidins being cyanidin. Total anthocyanidin concentration alone does not fully explain skin color intensity. Our findings contrast with those reported by Liang et al. [22], who claimed no anthocyanins were detected in green-yellow cultivars.

Anthocyanidin composition showed a closer association with skin color than its total content. Yellowish-green grapes primarily contained cyanidin- and delphinidin- derived compounds, with cyanidin accounting for more than 80% of total anthocyanidins. Pinkish grape accessions contained three to four anthocyanidin types, with cyanidin consistently serving as the dominant component. In contrast, purplish grapes typically harbored all five major anthocyanidin species—delphinidin, cyanidin, petunidin, peonidin, and malvidin—among which malvidin was the predominant compound, frequently accounting for over 50% of total anthocyanidin content.

### 3.4. Multivariate Analysis of Interactions Among Anthocyanidins, pH, and Skin Color Phenotypes

**Figure 5:**
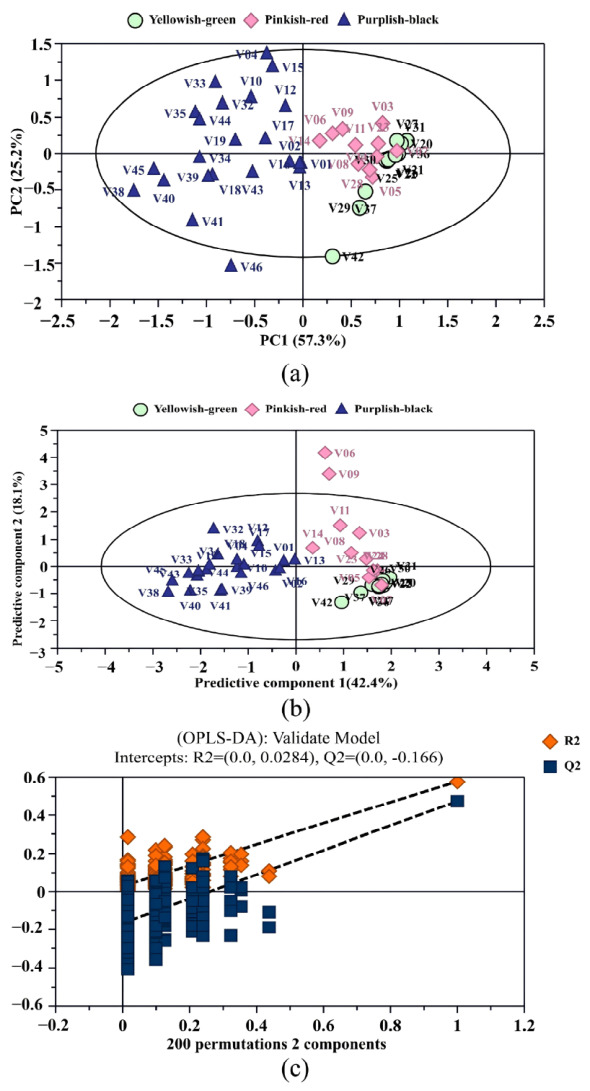
Multivariate analysis of anthocyanidin profiles and berry skin color. (**a**) PCA score plot based on the relative proportions of anthocyanidins. (**b**) Orthogonal partial least squares-discriminant analysis (OPLS-DA) score plot showing discrimination among grape skin color groups. (**c**) Permutation test validating the OPLS-DA model.

PCA based on relative proportions (%) clearly separated purplish grapes from yellowish-green and pink grapes, whereas partial overlap was observed between the latter two groups (Figure 5a), indicating distinct anthocyanidin profiles in purplish grapes. OPLS-DA further enhanced group discrimination, yielding a model with good explanatory and predictive performance (R^2^Y = 0.626, Q^2^ = 0.561). After 200 permutation tests, the intercepts of R^2^ and Q^2^ were 0.0284 and −0.166, respectively, further confirming the reliability of the model. (Figure 5b,c).

**Figure 6:**
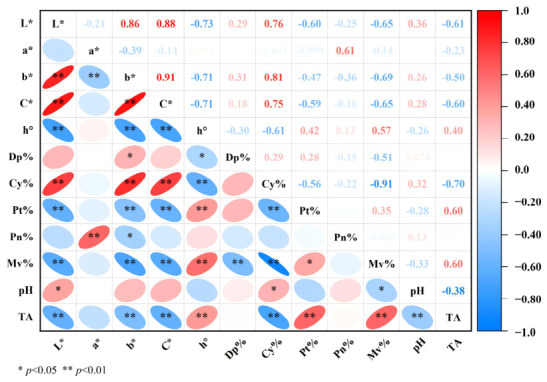
Correlation analysis among grape skin color parameters, skin homogenate pH, and anthocyanidin composition. Heatmap showing Pearson correlation coefficients between color parameters (L*, a*, b*), pH, and the relative proportions of different anthocyanidin-derived compounds (* *p* < 0.05; ** *p* < 0.01). Dp, delphinidin; Cy, cyanidin; Pt, petunidin; Pn, peonidin; Mv, malvidin; TA, total anthocyanidins.

Variable Importance in Projection (VIP) analysis was conducted, with compounds exhibiting VIP values exceeding 1.0 defined as significant contributors. This analysis identified cyanidin-, malvidin-, and peonidin-derived compounds as the main contributors to skin color differentiation (Table S2). Pearson correlation analysis showed L* values were positively correlated with the proportion of cyanidin-derived compounds and negatively correlated with the proportions of petunidin- and malvidin-derived compounds, as well as with total anthocyanidin content (Figure 6). In addition, a* values showed a significant positive correlation with peonidin-derived compounds in our results, suggesting their key role in red hue expression. This finding contrasts with that reported by Chen et al. [23], in which a delphinidin-derived compound (delphinidin-3-O-(6-O-Malonyl-β-D-glucoside) exhibited a significant positive correlation with a*, whereas higher proportions of malvidin- and petunidin-derived compounds were associated with blue-purple hues. As the key material basis underlying fruit coloration, the compositional proportions of different anthocyanidins directly influence hue differentiation in fruits.

Grape skins enriched in cyanidin-derived compounds tended to exhibit higher skin homogenate pH values and lower total anthocyanidin contents and generally lacked intense red-purple coloration. This pattern is consistent with pH-dependent structural transformations of anthocyanidins that reduce color intensity under relatively high pH conditions. Under low-pH conditions, anthocyanidins mainly exist as stable flavylium cations, exhibiting a characteristic red color. In yellowish-green grape cultivars, anthocyanidins were detected but failed to produce red-purple pigmentation.

### 3.5. Structural Diversity of Anthocyanins in Representative Grape Cultivars

**Figure 7:**
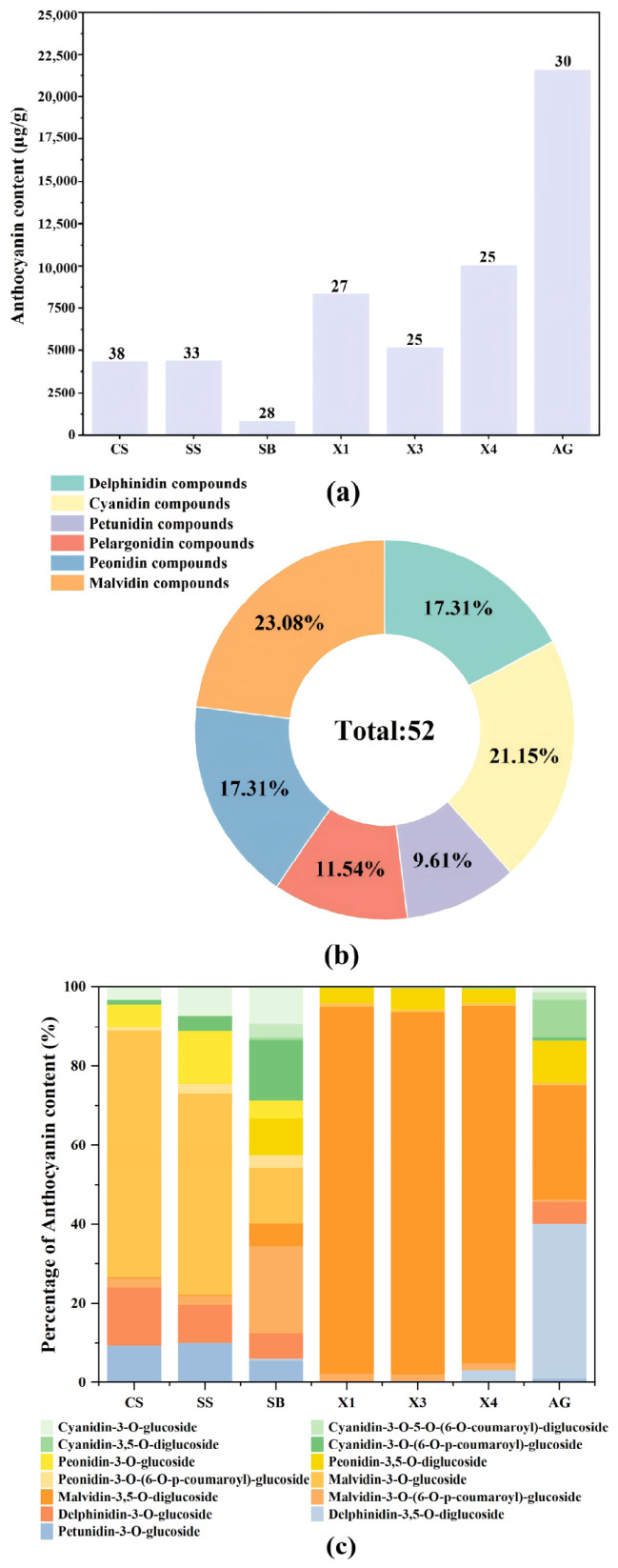
Anthocyanin composition and structural characteristics in representative grape cultivars. (**a**) Total anthocyanin contents of selected grape cultivars. (**b**) Ratio between the number of detected anthocyanidin derivatives and the total number of anthocyanins (*n* = 52). (**c**) Stacked percentage plot of major anthocyanins after excluding compounds with relative contents below 0.1%. CS, Cabernet Sauvignon; SS, Lanbaoshi; SB, Summer Black; X1, Xiangci No. 1; X3, Xiangci No. 3; X4, Xiangci No. 4; AG, Adenostoma grape.

In the grape cultivars examined, anthocyanidins were mainly present as glycosylated anthocyanins, consistent with previous observations in grape and other plant tissues [24,25]. Anthocyanidin glycosylation might exert a certain influence on the color expression of grape berry skins [26,27,28]. To assess whether structural variations in anthocyanins correlate with grape skin color, seven purple-black accessions with sufficiently high anthocyanin levels were selected from the 46 germplasm resources. Yellowish-green accessions were excluded due to their relative low anthocyanin content and limited anthocyanin type with over 80% being Cyanidin, which precluded reliable detection. The selected accessions represent three major genetic backgrounds (*V. vinifera* cultivars, interspecific hybrids and wild grape relatives) and include both commercially cultivated varieties and regionally characteristic landraces. Their total anthocyanin contents are presented in Figure 7a.

A total of 52 anthocyanin compounds were identified using UPLC-MS/MS, among which 17 major components collectively accounted for over 99.5% of the total anthocyanin pool (Figure 7b,c). Notably, while pelargonidin was undetectable via the aforementioned HPLC-based method, UPLC–MS/MS successfully identified trace levels of pelargonidin-derived anthocyanins. This discrepancy is likely attributable to the superior analytical sensitivity of UPLC–MS/MS, coupled with potential minor losses of analytes during the acid hydrolysis step employed for anthocyanidin aglycone determination. In addition, several rarely reported anthocyanins (e.g., sambubioside, sophoroside, xyloside) were also detected. This finding is primarily attributable to the high sensitivity of and the targeted detection approach based on 93 authentic anthocyanin standards, which facilitated reliable qualification and quantification of low-abundance compounds. Although the relative abundances of these rare anthocyanins were generally low, suggesting a less direct contribution to static berry skin color, their presence broadens the known chemical diversity of grape anthocyanins.

Cabernet Sauvignon and Lanbaoshi (*V. vinifera* L.) were mainly composed of monoglycosylated and acylated anthocyanins, with malvidin-3-O-glucoside as the predominant compound. Summer black (*V. vinifera* × *V. labrusca*) showed a similar structural pattern but was dominated by malvidin-3-O-(6-O-p-coumaroyl)-glucoside. In contrast, Xiangci No. 1, Xiangci No. 3, Xiangci No. 4, and Adenostoma grape, all belonging to East Asian grape species, predominantly accumulated diglycosylated anthocyanins, with malvidin-3,5-O-diglucoside as the major component. The observed species-dependent anthocyanin profiles are consistent with findings reported in previous studies [29,30]. Among the seven purple-black accessions selected for UPLC-MS/MS analysis, the anthocyanins identified herein were predominantly malvidin derivatives conjugated with various glycosyl moieties. Notably, acid hydrolysis of these compounds consistently yielded malvidin as the sole detectable aglycone in these selected seven cultivars, which is in strong agreement with the HPLC-based anthocyanidin results presented in the preceding section.

**Figure 8:**
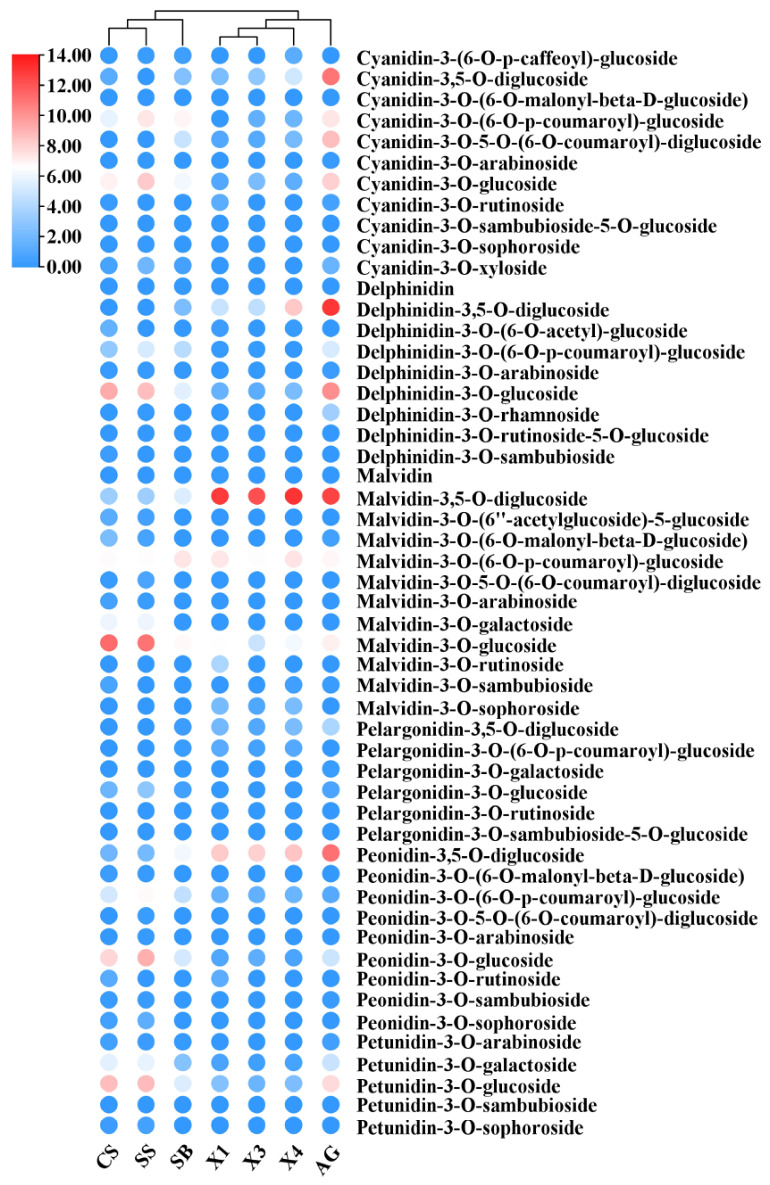
Heatmap of the relative abundance of major anthocyanins across grape cultivars. Heatmap showing the relative abundance of major anthocyanins across grape cultivars. CS, Cabernet Sauvignon; SS, Lanbaoshi; SB, Summer Black; X1, Xiangci No. 1; X3, Xiangci No. 3; X4, Xiangci No. 4; AG, Adenostoma grape.

Based on Figure 8, the selected grape accessions were divided into two groups for intergroup comparison. Group 1 included Cabernet Sauvignon, Lanbaoshi, and Summer Black (*V. vinifera* cultivars and interspecific hybrids), whereas Group 2 consisted of four grape wild relatives: Xiangci No. 1, Xiangci No. 3, Xiangci No. 4, and Adenostoma grape. Both groups displayed purplish-black skin coloration and comparably high anthocyanidin levels. However, cultivars in Group 2 frequently exhibited secondary glycosylation at the C-5 hydroxyl position of anthocyanidin aglycones, forming anthocyanin diglucoside derivatives. For chromatic measurements, ten berries were randomly selected from each accession, resulting in a total of 30 berries in Group 1 and 40 berries in Group 2. Since the color parameter data failed to meet the normality assumption (Shapiro–Wilk test, *p* < 0.05), a non-parametric Mann–Whitney U test was employed (Table S3), which does not require homogeneity of variance. The test results indicated a general trend toward lower L*, a*, and b* values in Group 2, consistent with reduced brightness and a slightly more bluish-purple hue. Among these color parameters, only the b* value showed a statistically significant difference between the two groups (*p* < 0.05), while no significant differences were observed for L* or a* values (Table S3).

## 4. Discussion

Although this study reveals the dominant roles of anthocyanin composition and structural modifications in determining berry skin color, several limitations should be acknowledged, some of which may confound the interpretation of pH-anthocyanidin-color relationships.

First, as noted above, skin color results from the synergistic action of multiple pigment classes; we did not quantify other pigments such as chlorophylls and carotenoids. This omission prevents us from precisely evaluating their masking or additive effects on anthocyanin-based coloration, which in turn complicates the interpretation of the pH–anthocyanidin–color relationships. The unmeasured pigments may alter the perceived color hue and intensity, potentially obscuring the true correlations between pH, anthocyanidin composition, and skin color.

Second, the 46 accessions were collected from different regions of China, and variations in growing environment (climate, soil, cultivation practices) and harvest time may have introduced confounding effects on skin homogenate pH, anthocyanin accumulation, and color parameters. Although we standardized fruit maturity using soluble solids content, interference from environmental heterogeneity cannot be completely excluded. These environmental factors may independently influence both pH levels and anthocyanidin profiles, making it challenging to isolate the specific effects of anthocyanin composition on skin color, and thereby complicating the interpretation of the pH–anthocyanidin–color relationships.

Therefore, future studies should conduct multi-year, multi-site replicated trials under uniform environmental conditions to further validate the conclusions drawn herein.

In this study, measured skin homogenate pH values (ranging from 3.36 to 4.63) exhibited significant correlations with color parameters. Consistent with previous literature [31], this relationship may be attributed to pH-mediated shifts in the protonation/deprotonation equilibrium of anthocyanidin aglycones. For instance, although yellowish-green grape accessions contained trace amounts of anthocyanidins, they did not exhibit visible red-purple coloration. This phenomenon is likely driven by two key factors: (i) relatively higher pH may convert the anthocyanidin pigments into colorless carbinol pseudo-bases or yellow retro-chalcones [32]; and (ii) the visual masking effect of other skin pigments (e.g., chlorophylls, carotenoids) cannot be overlooked. Peonidin-derived anthocyanins were associated with red hues, while malvidin-derived compounds correlated with blue-purple coloration.

At the level of structural modifications, distinct glycosylation and acylation patterns were observed among *V. vinifera* cultivars, interspecific hybrids and wild grape relatives. Under the experimental conditions of this study (i.e., high anthocyanin content and minimal pH differences), the contribution of these structural variations to visually perceived skin color was negligible, with only subtle differences detectable via colorimetric analysis. However, the data suggest a potential association between higher diglucoside content in wild grape relatives and significantly lower b* values (indicating a bluer hue) [26]. Inter-group differences in glycosylation patterns exhibited bimodal distribution: *V. vinifera* accessions were dominated by monoglucosides, whereas wild grape relatives primarily accumulated diglucosides, with few intermediate types observed. Genetically, this polarization reflects functional divergence of flavonoid 5-O-glucosyltransferase (5-GT) genes [33]. During evolution, *V. vinifera* underwent a loss-of-function mutation in the 5-GT gene, restricting anthocyanin synthesis to 3-O-monoglucosides. In contrast, wild grape relatives have retained functional 3-GT and 5-GT enzymes, enabling efficient accumulation of 3,5-O-diglucosides [34,35]. Under the limitation of bimodal glycosylation distribution, mono/diglucosides may have potential effects on skin color, but their specific impacts cannot be accurately evaluated at present. The higher proportion of diglucosides in wild grape relatives may not only serve as the structural basis for their bluer hue but also represent an important evolutionary strategy for enhancing pigment stability and environmental adaptability.

It is important to note that the germplasm used in this study was predominantly collected from East Asia (primarily China) and excluded North American grape species such as *Vitis rotundifolia*, in which delphinidin and other pigments may play more dominant roles. Consequently, the conclusions concerning the relationship among anthocyanidin composition, pH, and skin color are specific to the examined accessions and should not be generalized to all grape species without further validation.

Given the bimodal distribution of glycosylation ratios in the available germplasm, it is currently not possible to accurately assess the quantitative effect of mono/diglucoside ratios on skin color. Additionally, several rarely reported trace anthocyanins were detected in this study, such as sambubiosides. Although these compounds contributed minimally to static skin color, they significantly expand the known chemical diversity of grape anthocyanins. They may enhance color stability in wild grape relatives through unique glycosylation patterns. Further studies are needed to investigate their specific functions. Future studies should (i) construct germplasm collections with continuous gradients of glycosylation ratios or conduct in vitro addition experiments to explore the impact of mono/di-glucoside ratios on pigment stability, particularly during juice and wine processing; and (ii) evaluate the potential transformation and color contribution of these trace anthocyanins under biotic/abiotic stress or during post-harvest processing. Moreover, to more precisely dissect the independent contributions of glycosylation and acylation to skin color, future research should include a wider range of accessions with moderate anthocyanin contents and diverse color gradients.

## 5. Conclusions

To our knowledge, this study represents one of the few systematic investigations that integrate berry color, skin homogenate pH, and anthocyanidin composition across a diverse panel of 46 grape accessions, including nine wild grape relatives. Skin color exhibited significant variation among accessions, and was objectively classified into three distinct groups—yellowish-green, pinkish-red, and purplish-black—based on CIE Lab* parameters. Total anthocyanidin content alone proved insufficient for evaluating berry skin color quality; instead, the composition and relative proportions of individual anthocyanidins showed a stronger association with color classification. Among the germplasm accessions investigated, cyanidin, malvidin, and peonidin derivatives were found to be strongly correlated with berry skin color variation. Furthermore, skin homogenate pH was associated with anthocyanidin stability and significantly associated with color intensity. The wild grape relatives displayed unique anthocyanin profiles that may serve as valuable genetic resources for breeding programs. Collectively, this comparative study enhances our understanding of berry skin color from a comprehensive chemical perspective and may assist grape breeders in selecting desired color traits and evaluating fruit quality. However, given the correlational nature of this study and potential confounding factors, the observed associations should be validated under controlled multi-year and multi-site conditions.

## Figures and Tables

**Table 1:** Mean values (±SD) and statistical significance of CIE Lab color parameters among different grape skin color groups.

Group	L*	a*	b*	C*	h°
A	34.8 ± 2.91 ^a^	−0.856 ± 0.660 ^c^	6.59 ± 1.90 ^a^	6.68 ± 1.92 ^a^	97.6 ± 5.34 ^c^
B	30.8 ± 2.73 ^b^	3.39 ± 1.24 ^a^	1.10 ± 1.52 ^b^	3.98 ± 1.33 ^b^	352 ± 8.44 ^a^
C	26.7 ± 1.47 ^c^	0.758 ± 0.984 ^b^	−1.36 ± 0.482 ^c^	1.83 ± 0.711 ^c^	292 ± 27.1 ^b^

Group A: yellowish-green (*n* = 12); Group B: pinkish-red (*n* = 11); Group C: purplish-black (*n* = 23). Different lowercase letters within a column (^a^, ^b^, ^c^) indicate significant differences among groups (*p* < 0.05) based on one-way ANOVA followed by Tukey’s HSD test.

**Table 2:** Retention times, calibration curves (0.1–50 mg∙L^−1^), correlation coefficients and blank test results for six anthocyanidins.

No.	Compounds	RT/min	Linear Equations		Correlation Coefficients (R^2^)	Blank
			a	b		
1	Dp	6.97	51,806.9	−4908.1	0.99992	^a^ N.D.
2	Cy	8.92	29,550.2	−3803.2	0.99991	N.D.
3	Pt	9.57	42,362.0	−4590.2	0.99991	N.D.
4	Pg	11.8	34,336.9	−5007.4	0.99992	N.D.
5	Pn	12.9	50,001.4	−5800.9	0.99991	N.D.
6	Mv	13.5	35,154.8	−4636.4	0.99992	N.D.

Dp, delphinidin; Cy, cyanidin; Pt, petunidin; Pg, pelargonidin; Pn, peonidin; Mv, malvidin. ^a^ N.D. indicates that the compound was not detected.

**Table 3:** LOD, LOQ, RSD of instrument precision (RSD_1_), the intra-day (RSD_2_) precision, and recoveries of six anthocyanidins in spiked samples.

No.	Compounds	LOD /mg∙L^−1^	LOQ /mg∙kg^−1^	RSD_1_/%	RSD_2_ ^b^/%	Recovery %		
						^a^ 10 mg∙L^−1^	1.0 mg∙L^−1^	0.5 mg∙L^−1^
1	Dp	0.0025	0.1660	0.44	0.56	100.5	119.1	90.12
2	Cy	0.0050	0.3340	1.2	0.79	99.90	102.9	87.46
3	Pt	0.0050	0.3340	0.45	1.0	106.4	99.04	109.6
4	Pg	0.0050	0.3340	0.43	/ ^c^	91.15	84.70	108.4
5	Pn	0.0050	0.3340	0.38	0.90	87.73	101.2	100.1
6	Mv	0.0050	0.3340	0.50	0.41	86.51	81.85	82.46

Dp, delphinidin; Cy, cyanidin; Pt, petunidin; Pg, pelargonidin; Pn, peonidin; Mv, malvidin. ^a^ indicates the spiked concentration at three levels: low (0.5 mg∙L^−1^), medium (1.0 mg∙L^−1^), and high (10 mg∙L^−1^). ^b^ RSD_2_ represents the relative standard deviation of each anthocyanidin content determined in the ‘Yeniang No. 2’ sample, by repeated injections of the prepared sample solution at 2 h intervals over a 24 h period. ^c^ Pelargonidin was not detected in the sample solution used for the stability test; therefore, its RSD_2_ value could not be calculated (marked as ‘/’ in the table).

## Data Availability

The original contributions presented in the study are included in the article/Supplementary Material; further inquiries can be directed to the corresponding author.

## Supplementary Materials

The following supporting information can be downloaded at: https://www.mdpi.com/article/10.3390/foods15122242/s1, Table S1: Information of grape samples used in this study; Table S2: Compounds with variable importance in projection (VIP) values; Table S3: Mann–Whitney U test of chromatic parameters among seven grape cultivars.

## Abbreviations

The following abbreviations are used in this manuscript:

CIE	International Commission on Illumination
HPLC	High Performance Liquid Chromatography
UPLC-MS/MS	Ultra Performance Liquid Chromatography-tandem Mass Spectrometry
ESI	Electrospray ionization
CUR	Curtain gas
DP	Declustering potential
CE	Collision energy
MWDB	Metware Database
MRM	Multiple Reaction Monitoring
PCA	Principal Component Analysis
OPLS-DA	Orthogonal Partial Least Squares Discriminant Analysis
SD	Standard Deviation
VIP	Variable Importance in the Projection
Dp	Delphinidin
Cy	Cyanidin
Pt	Petunidin
Pn	Peonidin
Mv	Malvidin
