# Supplementary material for: Anthocyanin Composition and pH Correlate with Berry Skin Color Across Diverse Grape Germplasm
Source: Foods. 2026 Jun 22;15(12):2242. doi: 10.3390/foods15122242 (PMC13297887; doi:10.3390/foods15122242)
Supplement: Supplementary file 1 [file foods-15-02242-s001.zip › foods-4368829-supplementary.pdf]

SUPPORTING INFORMATION

Anthocyanin Composition and pH Correlate with Berry Skin Color across Diverse Grape Germplasm

Fei Peng, Weichu Ouyang, Wenting Chen, Feixiong Luo, Yanshuai Xu, Guoshun Yang, Jun Tan*

College of Horticulture, Hunan Agricultural University, Changsha, 410128, China

* Corresponding Author. Email address: junjuntan@163.com

Tel: +86-731-84618171

Table S1. Information of grape samples used in this study.

Sample No.	Species	Cultivar	Soluble solids content (°Brix)	Collection site
V01	*Vitis labrusca* × *Vitis vinifera*	Early-ripening sport of Summer Black, Anhui	20.0±1.94	Changsha, Hunan
V02	*V. labrusca* × *V. vinifera*	Early-ripening sport of Summer Black, Donghai	18.7±0.900	Changsha, Hunan
V03	*V. vinifera*	Muscat Hamburg	17.9±1.01	Changsha, Hunan
V04	*V. vinifera*	Heimeiren (Manicure Finger O.P)	21.8±1.56	Changsha, Hunan
V05	*V. vinifera*	Red sport of Muscat of Alexandria	18.2±0.750	Changsha, Hunan
V06	*V. vinifera*	Red Globe	19.0±1.18	Wenshan, Yunnan
V07	*V. vinifera*	Rosario Rosso	18.3±0.990	Changsha, Hunan
V08	*V. vinifera*	Ruidu Hongmei	18.0±2.27	Changsha, Hunan
V09	*V. vinifera*	Crimson Seedless	17.8±0.710	Changsha, Hunan
V10	*V. vinifera* × *V. labrusca*	Baoguang	18.0±0.640	Changsha, Hunan
V11	*V. vinifera*	Bolicui	22.3±0.770	Tulufan, Xinjiang

Sample No.	Species	Cultivar	Soluble solids content (°Brix)	Collection site
V12	*V. vinifera* × *V. labrusca*	Chunguang	18.0±1.17	Changsha, Hunan
V13	*V. vinifera* × *V. labrusca*	Hutai No.8	17.6±1.13	Changsha, Hunan
V14	*V. vinifera* × *V. labrusca*	Fenghou	17.3±0.990	Changsha, Hunan
V15	*V. vinifera*	Zitian	18.8±2.33	Changsha, Hunan
V16	*V. vinifera* × *V. labrusca*	Early-ripening sport of Summer Black, Shangfeng	20.7±1.65	Changsha, Hunan
V17	*V. vinifera* × *V. labrusca*	Kyoho	19.3±2.67	Changsha, Hunan
V18	*V. vinifera*	Lanbaoshi	18.2±0.890	Yuncheng, Shanxi
V19	*V. vinifera* × *V. labrusca*	Summer Black	18.1±1.27	Zhengzhou, Henan
V20	*V. vinifera* × *V. labrusca*	Tianshan	19.1±0.810	Changsha, Hunan
V21	*V. vinifera*	Muscat of Alexandria	18.5±1.54	Changsha, Hunan
V22	*V. vinifera* × *V. labrusca*	Zuijinxiang	19.5±2.32	Changsha, Hunan
V23	*V. vinifera*	Manicure Finger	19.2±1.74	Changsha, Hunan
V24	*V. vinifera*	Chang'e Finger	18.0±2.06	Changsha, Hunan
V25	*V. vinifera* × *V. labrusca*	Ruiduxiangyu	18.0±1.96	Changsha, Hunan
V26	*V. vinifera*	Ruidukemei	17.5±1.45	Changsha, Hunan
V27	*V. vinifera*	Xiangfei	17.9±0.890	Changsha, Hunan
V28	*V. vinifera* × *V. labrusca*	Nina Queen	20.0±1.98	Changsha, Hunan
V29	*V. vinifera*	Thompson seedless	18.2±1.18	Tulufan, Xinjiang
V30	*V. vinifera* × *V. labrusca*	Jinguang	18.4±1.37	Changsha, Hunan

Sample No.	Species	Cultivar	Soluble solids content (°Brix)	Collection site
V31	*V. vinifera* × *V. labrusca*	Shine Muscat	17.8±1.23	Changsha, Hunan
V32	*V. vinifera*	Yan 73	16.3±1.04	Changsha, Hunan
V33	*V. vinifera*	Cabernet Gernischt	18.8±0.90	Zhangjiakou, Hebei
V34	*V. vinifera*	Cabernet Sauvignon	19.9±2.21	Zhangjiakou, Hebei
V35	*V. vinifera*	Marselan	21.9±0.990	Zhangjiakou, Hebei
V36	*V. vinifera*	Chardonnay	16.5±1.23	Changsha, Hunan
V37	*V. vinifera*	Ugni Blanc	15.1±1.18	Zhangjiakou, Hebei
V38	*V. davidii*	Gaoshan Spine Grape	16.8±1.90	Zhijiang, Hunan
V39	*V. davidii*	Xiangci No.1	12.6±0.950	Changsha, Hunan
V40	*V. davidii*	Xiangci No.4	11.0±0.600	Changsha, Hunan
V41	*V. davidii*	Xiangci No.3	11.8±1.73	Changsha, Hunan
V42	*V. davidii*	Xiangci No.2	14.6±0.740	Changsha, Hunan
V43	*V. heyneana*	Yeniang No.2	11.0±1.30	Guilin, Guangxi
V44	*V. amurensis*	Amur Grape	16.6±2.30	Yanqing, Beijing
V45	*V. vinifera* × *V. labrusca*	Lingfenghong	19.1±1.57	Shenyang, Liaoning
V46	*V. adenoclada*	Adenoclada grape	17.3±1.95	Hengyang, Hunan

Table S2. Compounds with variable importance in projection (VIP) values.

Number	Var ID (Primary)	VIP
1	Cy%	1.27878
2	Mv%	1.22481
3	Pn%	1.19503
4	Pt%	0.811005
5	pH	0.734988
6	Dp%	0.488406

Cy, cyanidin; Mv, malvidin; Pn, peonidin; Pt, petunidin; Dp, delphinidin.

Table S3. Mann-Whitney U test of chromatic parameters among seven grape cultivars.

Parameter	Group	Sample size	Median	Interquartile range (Q1~Q3)	U	Z-value	*p*-value
L*	1	30	26.8	26.0~27.7	556	-0.522	0.602
	2	40	26.8	24.2~28.2			
a*	1	30	0.375	-0.313~1.09	545	-0.659	0.510
	2	40	0.320	-0.250~0.830			
b*	1	30	-0.950	-1.12~-0.845	371	-2.72	0.007*
	2	40	-1.55	-1.90~-0.840			
C*	1	30	1.38	1.06~1.71	477	-1.47	0.143
	2	40	1.62	0.890~2.08			
h°	1	30	286	257~316	535	-0.771	0.440
	2	40	301	280~322			

Differences were considered significant at $p < 0.05$; only b* showed a significant difference ($p = 0.007$).
